# Thoracoscopic endoscopic cooperative surgery for gastric tube cancer after esophagectomy: a case report

**DOI:** 10.1093/jscr/rjae034

**Published:** 2024-02-03

**Authors:** Toshikatsu Tsuji, Koichi Okamoto, Hiroto Saito, Mari Shimada, Hideki Moriyama, Jun Kinoshita, Hajime Takatori, Noriyuki Inaki

**Affiliations:** Department of Gastrointestinal Surgery/Breast Surgery, Kanazawa University Graduate School of Medical Science, 13-1 Takaramachi, Kanazawa, Ishikawa 920-8641, Japan; Department of Gastrointestinal Surgery/Breast Surgery, Kanazawa University Graduate School of Medical Science, 13-1 Takaramachi, Kanazawa, Ishikawa 920-8641, Japan; Department of General and Digestive Surgery, Kanazawa Medical University Hospital, 1-1 Digaku, Uchinadamachi, Ishikawa, Kahoku, Ishikawa 920-0293, Japan; Department of Gastrointestinal Surgery/Breast Surgery, Kanazawa University Graduate School of Medical Science, 13-1 Takaramachi, Kanazawa, Ishikawa 920-8641, Japan; Department of Gastrointestinal Surgery/Breast Surgery, Kanazawa University Graduate School of Medical Science, 13-1 Takaramachi, Kanazawa, Ishikawa 920-8641, Japan; Department of Gastrointestinal Surgery/Breast Surgery, Kanazawa University Graduate School of Medical Science, 13-1 Takaramachi, Kanazawa, Ishikawa 920-8641, Japan; Department of Gastrointestinal Surgery/Breast Surgery, Kanazawa University Graduate School of Medical Science, 13-1 Takaramachi, Kanazawa, Ishikawa 920-8641, Japan; Department of Gastroenterology, Kanazawa University Graduate School of Medical Science, 13-1 Takaramachi, Kanazawa, Ishikawa 920-8641, Japan; Department of Gastrointestinal Surgery/Breast Surgery, Kanazawa University Graduate School of Medical Science, 13-1 Takaramachi, Kanazawa, Ishikawa 920-8641, Japan

**Keywords:** esophageal cancer, esophagectomy, gastric tube cancer, thoracoscopic endoscopic cooperative surgery

## Abstract

The incidence of gastric tube cancer (GTC) is increasing due to the improved prognosis of patients after esophagectomy for esophageal cancer. Total resection of the gastric tube is expected to be curative for patients with GTC. However, several studies have reported that this procedure is associated with high mortality and morbidity rates. We here present a case of GTC without lymph node metastasis in a patient who underwent partial resection of a gastric tube via thoracoscopic-endoscopic cooperative surgery. No postoperative complications or recurrence was observed. This procedure is a favorable and minimally invasive procedure for GTC without lymph node metastasis.

## Introduction

Prognostic improvement in patients with esophageal cancer who have undergone esophagectomy has caused increased gastric tube cancer (GTC) incidence [1, 2]. Patients with GTC undergo total gastric tube resection with lymphadenectomy. However, this surgical treatment is highly invasive and is associated with high mortality and morbidity [3].

Herein, we report a case of GTC without lymph node metastasis that was treated with partial gastric tube resection via thoracoscopic endoscopic cooperative surgery, and describe the patient’s outcomes.

## Case report

A 69-year-old man had undergone thoracoscopic subtotal esophagectomy reconstruction through the posterior mediastinal route, with three-field lymphadenectomy, for thoracic esophageal cancer 13 years previously. The pathological diagnosis was T2N0M0-pStageII.

**Figure 1:**
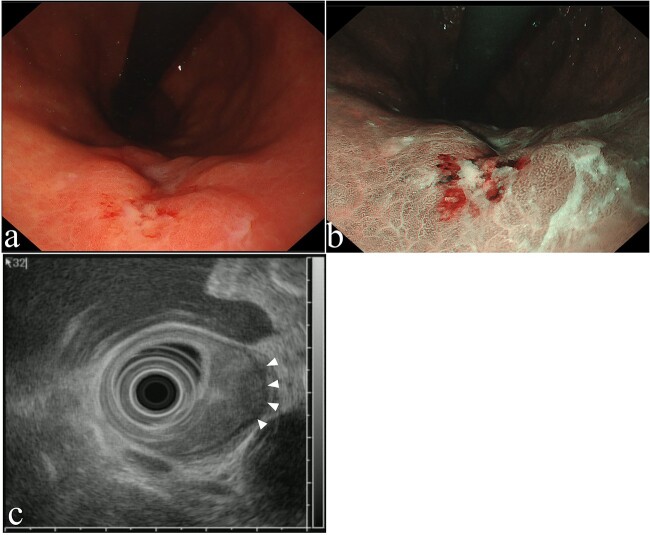
Upper gastrointestinal endoscopy findings. (a) 15-mm 0-IIc lesion, (b) narrow-band imaging. (c) Endoscopic ultrasound image revealed a low-echoic lesion with invasion of the muscularis propria layer (arrowheads).

The patient visited our institution with epigastric pain. Upper gastrointestinal endoscopy revealed a 15-mm 0-IIc lesion in the middle of the gastric body (Fig. 1a and b). Biopsy identified adenocarcinoma. Endoscopic ultrasonography revealed that the tumor had invaded the submucosal layer or muscularis propria (Fig. 1c). The primary lesions could not be detected on computed tomography, and no lymph node or distant metastases were observed. The patient had a history of hemiplegia secondary to cerebral hemorrhage and had a poor performance status [Eastern Cooperative Oncology Group performance status (ECOG-PS) 2]. In locally advanced GTC in which muscularis propria invasion is suspected, total gastric tube resection with lymphadenectomy may be ideal, but in a patient with a poor general condition, such as ECOG-PS 2, and a relatively early stage cancer, this approach may be highly invasive. The patient refused total gastric tube resection. Therefore, we planned partial resection via thoracoscopic endoscopic cooperative surgery, as a minimally invasive procedure, with the patient’s consent (Supporting information; Video S1).

**Figure 2:**
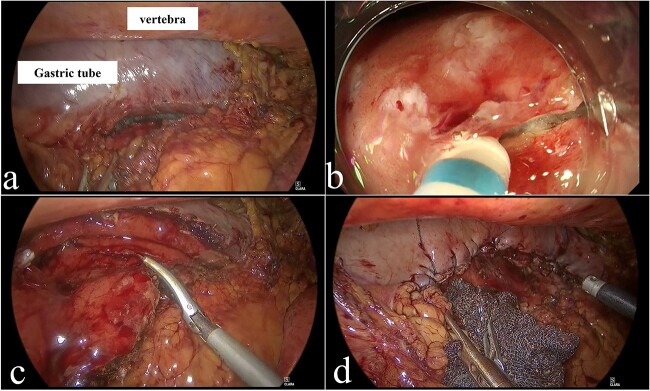
Operative findings. (a) Adhesion detachment was performed to expose the gastric tube. (b) Submucosal dissection using Dual Knife. (c) Resection of specimen using ultrasonic energy device. (d) Complete closure of wall defect using barbed sutures.

After general anesthesia induction, the patient was placed in a semi-prone position. Four ports were placed, as follows: a 12-mm port for the camera at the ninth intercostal shoulder blade line; a 5-mm port for the operator’s left working hand at the seventh intercostal posterior axillary line; a 12-mm port for the operator’s right working hand at the fifth intercostal middle axillary line; and a 5-mm port for assistance at the sixth intercostal anterior axillary line. Pneumomediastinal pressure was maintained at 8 mmHg. We exposed the gastric tube (Fig. 2a) and identified the tumor location endoscopically. Tumor margins were marked on the mucosa. After submucosal injection, a circumferential mucosal incision was made around the lesion using a Dual Knife (Olympus, Tokyo, Japan) (Fig. 2b). Submucosal dissection was performed using the Dual Knife and a full-thickness incision was made. Under thoracoscopic guidance, the specimen was resected using a harmonic scalpel (Ethicon; Somerville, New Jersey, USA) (Fig. 2c). The resected specimen was placed inside a plastic bag and extracted from the intercostal space. The wall defect was closed with continuous full-layer barbed sutures (3-0 V-LOC™ 180, Medtronic plc, Dublin, Ireland) (Fig. 2d). The operative time was 216 min, and almost no bleeding occurred. The patient was discharged on the 12th postoperative day, without complications.

**Figure 3:**
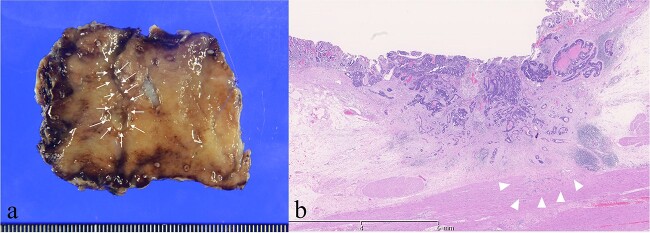
Histopathological findings. (a) Resected specimen. 13 × 8 mm 0-IIc lesion (arrows). (b) The tumor invaded muscularis propria (arrowheads) (Hematoxylin and Eosin staining).

The pathological findings were advanced GTC; 0–IIc, 13 × 8 mm, tub2 > por2 > tub1, INFβ, Ly1a, V1a, pT2(MP), pPM0, and pDM0 (Fig. 3a and b). The patient is currently under observation at our hospital. One and a half years after the surgery, no recurrence has been observed.

## Discussion

The use of thoracoscopic endoscopic cooperative surgery in a patient with GTC without lymph node metastases is a minimally invasive, function-preserving treatment.

Total gastric tube resection with lymphadenectomy and colonic or jejunal interposition is the standard GTC treatment. GTC surgery is associated with high mortality and morbidity rates. Akita *et al*. have reported five cases of total resection of the gastric tube, of which one patient died of postoperative complications [4], while Sugiura *et al*. reported five such patients with a 100% surgical morbidity rate [3]. The post-total gastric tube resection complication rate has been reported to be 83% [5]. Total gastric tube resection may be unnecessarily invasive in some patients.

Laparoscopic endoscopic cooperative surgery was a surgical technique reported by Hiki *et al*. as a procedure combining laparoscopic gastric resection with endoscopic submucosal dissection for partial resection of gastric tumors with appropriate and minimal surgical resection margins [6]]. In the present case, the patient had GTC with submucosal or muscularis propria invasion without lymph node metastasis. Furthermore, he had a poor general condition (ECOG-PS 2). Therefore, we considered that partial resection of the gastric tube was reasonable and safe for this patient. No intraoperative or postoperative complications were observed, and the patient was satisfied.

Recently, endoscopic submucosal dissection (ESD) for early GTC has been reported to be safe and to result in good long-term outcomes [7]. In the present case, ESD was not indicated because of GTC with submucosal or muscularis propria invasion. We considered thoracoscopic endoscopic cooperative surgery to be a good indication for locally advanced GTC that could not be removed by ESD. Thoracoscopic endoscopic cooperative surgery is a local control treatment that is not indicated for GTC with lymph node metastasis. Therefore, detecting GTC early to enable minimally invasive treatments is crucial. The mean interval between esophagectomy and GTC diagnosis has been reported to be 55.8 months [5]. Therefore, long-term endoscopic follow-up is essential. In our case, GTC was diagnosed ˃13 years after esophagectomy. Upper gastrointestinal endoscopy is recommended every 2 years for at least 10 years after esophagectomy [8].

The incidence of GTC in older patients is expected to increase because of the improved survival of patients with esophageal cancer using esophagectomy. Therefore, selecting the treatment for GTC based on the patient’s general condition and degree of progress is important. We believe that cooperative thoracoscopic–endoscopic surgery could be a treatment option for patients with GTC without lymph node involvement in future.

In conclusion, partial gastric tube resection via thoracoscopic endoscopic cooperative surgery may be a minimally invasive and safe procedure for patients with GTC without lymph node metastasis. In particular, this treatment strategy is acceptable for GTC patients with poor PS and no lymph node metastasis because it balances surgical invasiveness and tumor curability.

## Supplementary Material

Supporting_Information_Video_rjae034
